# Building the future of synthetic organ manufacturing and healthcare

**DOI:** 10.1111/cpr.13497

**Published:** 2023-05-21

**Authors:** Qi Zhou

**Affiliations:** ^1^ State Key Laboratory of Stem Cell and Reproductive Biology Institute of Zoology, Chinese Academy of Sciences Beijing China; ^2^ University of Chinese Academy of Sciences Beijing China

Organ reconstruction and manufacturing is a field that aims to create functional organs for transplantation or therapeutic purposes. The development of this field has been driven by rapid advances in various technologies, including three‐dimensional (3D) bioprinting, organs‐on‐chips, organoids, stem cell reprogramming, genome editing and artificial intelligence (AI).

Take, for example, the development of organoids and organs‐on‐chips, which has completely revolutionized the way scientists study organ development, disease progression and drug effects in vitro. 3D bioprinting, which can produce tissues and organs with customized shapes, sizes and functions, has also made it possible to create complex structures with high precision and accuracy, including livers, kidneys, hearts, ears and skin grafts. Bioprinted tissues and organs may soon be used for transplantation on demand in the clinic. Furthermore, manipulation of cell fates and genomes with the latest techniques in reprogramming, genome editing and AI‐based computational modelling has allowed us to unlock the hidden potential within human cells and produce new human cell types with new capabilities. Genome editing of patient‐derived cells to correct any underlying defects could also ensure that synthetic organs are created with improved functionality and compatibility with the recipient's body. Advancements like these have paved the way for new biomedical applications such as personalized medicine and regenerative therapies.

In this special issue on Organ Reconstruction and Manufacturing, we have assembled a corpus of the latest studies that shed light on the cutting‐edge progress and challenges in this field. Our special issue highlights the most significant new findings, technologies and therapies that are emerging in this burgeoning field, providing a platform for experts in the field to share their thoughts and predict future trends. The topics covered in this special issue include leading‐edge technologies like 3D bioprinting, organs‐on‐chips and organoids, as well as genetically engineered cells and animals—all of which are critical pieces in the quest for regenerative medicine. If this dream is realized, scientists may well be able to use patient‐derived cells to complete drug testing at warp speed, and make it possible to create synthetic organs for transplantation, potentially addressing the shortage of donor organs on a global scale.

As we rapidly make technological progress towards this dream, a final piece in the puzzle is the development of ethical guidelines and manufacturing standards to ensure that the production and applications of synthetic organs remain safe, ethical and properly regulated. The creation of synthetic organs raises many ethical questions, including issues related to patient privacy, informed consent and accessibility to healthcare. Therefore it is crucial for us to establish ethical guidelines and manufacturing standards in a collaborative manner, based on multinational and global consensus, to regulate the proper development and use of synthetic organs. We believe our efforts in building such frameworks will help ensure that the benefits of technological breakthroughs in organ reconstruction and manufacturing are maximized, while minimizing potential risks and negative consequences for all of humanity.

In conclusion, synthetic organ reconstruction and manufacturing is a rapidly evolving field that has the potential to revolutionize the healthcare industry. I believe recent advancements in biotechnology, computational methods, therapeutic applications, ethics and standards will pave the way for a quantum leap in innovation for the field in the near future.

